# Rapid synthesis of hybrid methylammonium lead iodide perovskite quantum dots and rich MnI_2_ substitution favouring Pb-free warm white LED applications†

**DOI:** 10.1039/c9na00330d

**Published:** 2019-06-07

**Authors:** Rajan Kumar Singh, Sudipta Som, Somrita Dutta, Neha Jain, Mei-Tsun Kuo, Jai Singh, Ranveer Kumar, Teng-Ming Chen

**Affiliations:** Department of Physics, Dr. Harisingh Gour Central University Sagar 470003 M. P. India ranveerssi@yahoo.com +91 88635723764 +91 88635731695; Department of Chemical Engineering, National Taiwan University Taipei Taiwan Republic of China; Department of Applied Chemistry, National Chiao Tung University 1001 University Road Hsinchu 30010 Taiwan tmchen@mail.nctu.edu.tw

## Abstract

We present a facile room temperature synthesis of CH_3_NH_3_Pb_1−*x*_Mn_*x*_I_3_ perovskite quantum dots (PQDs) substituting manganese (Mn^2+^) at the lead (Pb^2+^) sites to minimize environmental pollution and make it commercially feasible. By varying the concentration of Mn^2+^ from 0 to 60%, the PQDs exhibit strong color tunability from red to orange color suggesting successful energy transfer due to Mn^2+^ inclusion. We observed a high external photoluminescence quantum yield (PLQY) of 98% for unsubstituted CH_3_NH_3_PbI_3_ and >50% for up to 15% Mn^2+^ substituted PQDs. The average lifetime of PQDs was found to shorten with increasing Mn^2+^ replacement. We demonstrate a white LED prototype by employing the CH_3_NH_3_Pb_1−*x*_Mn_*x*_I_3_ PQDs with green QDs on a blue LED chip. The CRI and CCT value varying from 92 to 80 and 5100 K to 2900 K, respectively, indicate the usability of the Mn^2+^ substituted PQDs as efficient warm white LEDs with a promising CRI and good stability.

## Abstract 2

We present a facile room temperature synthesis of CH_3_NH_3_Pb_1−*x*_Mn_*x*_I_3_ perovskite quantum dots (PQDs) substituting manganese (Mn^2+^) at the lead (Pb^2+^) sites to minimize environmental pollution and make it commercially feasible.

## Graphical abstract

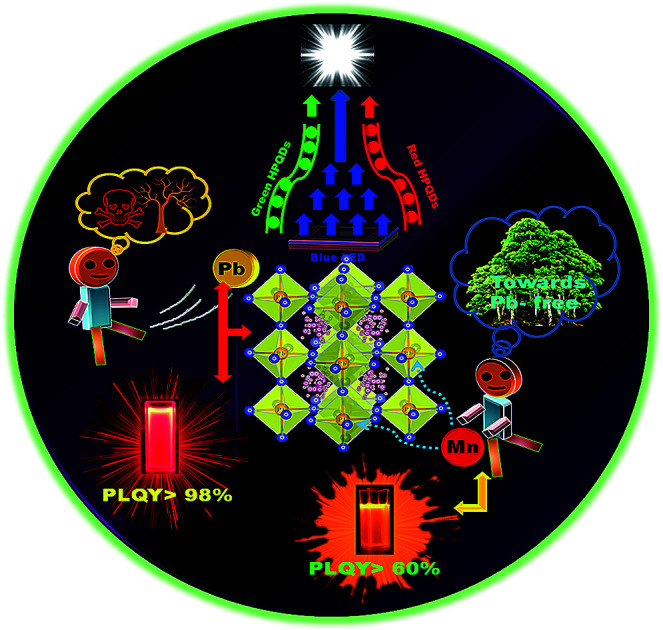

## Introduction

The discovery of hybrid perovskite quantum dots (HPQDs) (CH_3_NH_3_PbX_3_, X = Cl, Br, I) has opened a new era of research and development in new generation lighting technology as quantum dot light emitting diodes (QDLEDs) and backlight applications owing to their superior narrow emission, high photoluminescence quantum yield (PLQY), wide color range, and long diffusion length with high absorption coefficients.^1–4^ Moreover, the combination of organic/inorganic characteristics and an easy solution based synthesis approach at low temperature imparts a unique property to these materials. However, the presence of toxic Pb^2+^ at the B site in an ABX_3_ structure has restricted its commercialization.^5,6^ The use of heavy metals, including lead in an electronic device has already been restricted in the European Union, and other countries are also planning to introduce similar regulations in the near future.^7^ Therefore, the development of Pb^2+^ free or less Pb^2+^ based HPQDs that retain the excellent features of the original PQDs is obligatory. Cation exchange or substitution of Pb with divalent cations, such as, Cu^2+^, Zn^2+^, Sn^2+^, and Mn^2+^, could be a promising approach to modulate the optical and electronic properties of HPQDs.^8,9^ The forbidden ^4^T_1_ → ^6^A_1_ transition of Mn^2+^ makes this cation a suitable dopant to act as an economical colour emitter as well as decreasing the toxic level of the Pb-based perovskites due to its intense orange emission which remains independent of the physical and electronic configuration of the host.^6,9^

The lead-free mixed halides CsSnX_3_ (X = Cl, Br, I) fabricated by Jellicoe and group had stability issues under ambient conditions and had a low PLQY (<10%) due to the easy oxidation of Sn(ii) to Sn(iv).^10^ Liu *et al.* reported the partial replacement of Pb^2+^ with Mn^2+^ in CsPb_1−*x*_Mn_*x*_Cl_3_ PQDs (*x* = 0.3 ≤ *x* ≤ 0.4) with maximum PLQYs up to 54% *via* a hot injection route.^11^ Additionally, Mn^2+^ doping was also reported with CsPbBr_3_, CsPbCl_3−*x*_Br_*x*_ and CsPbI_3_ inorganic perovskite QDs by different groups.^12,13^ On the other hand, no reports are found on Pb substituted HPQDs and most of the studies were found on MAPbBr_3_ QDs due to their high stability and PLQY. The first solution based HPQDs were fabricated by the Pérez-Prieto group in 2014 with a PLQY of 20%.^14^ After that, Zhang *et al.* reported CH_3_NH_3_PbBr_3_ HPQDs in 2015 with an absolute quantum yield of up to 70% with the modification of the ligands in a room temperature process^15^ and recently the PLQY reached up to 100% by a spray synthesis route. However, obtaining high QY with CH_3_NH_3_PbI_3_ is still challenging with the maximum being 56% due to the higher sensitivity of iodine to moisture and air.^16,17^ Therefore, we need to work on increasing the PLQY for red QDs that can be used for red LEDs and perovskite quantum dot solar cells because for photovoltaic devices a MAPbI_3_ based material was found to be the best absorber.

To the best of our knowledge, Pb^2+^ substitution with Mn^2+^ in CH_3_NH_3_PbI_3_ PQDs by a room temperature synthesis method has not been reported to date. Therefore, in the present work, we present a novel approach to obtain CH_3_NH_3_PbI_3_ HPQDs with a PLQY of up to 98% and CH_3_NH_3_Pb_1−*x*_Mn_*x*_I_3_ HPQDs *via* Mn^2+^ substitution to reduce the toxicity of PQDs. We further demonstrate a white LED prototype by employing the as-prepared best CH_3_NH_3_Pb_1−*x*_Mn_*x*_I_3_ PQDs as color conversion materials, with green QDs on a blue LED chip to prove the probable commercialization of the present materials in the future for general lighting applications such as QDLEDs and backlight systems.

## Experimental section

### Synthesis methods

#### Materials required for synthesis

Methylamine (CH_3_NH_3_, 40% solution with water) was purchased from Merck, India. Methanol (CH_3_OH), lead(ii) iodide (PbI_2_, 98% Sigma, USA), hydroiodic acid, dimethyl formamide, DMF (anhydrous, 99.8%), toluene (anhydrous, 99.8%), oleic acid (≥99% (GC) and oleylamine (technical grade, 70%) from Sigma Aldrich were used for the synthesis of perovskite QDs.

#### Synthesis of methylamine iodide (MAI)

In a 250 ml round bottom flask, 20 ml methylamine, 8 ml methanol and 30 ml hydroiodic acid were mixed at 0 °C in an ice bath setup with continuous stirring for 2 h. The obtained solution was placed in a vacuum oven at 60 °C for 24 h to remove all the solvents. The final product was washed two times with diethyl ether to get the MAI salt and stored at dry place.

#### Synthesis of CH_3_NH_3_PbI_3_ and CH_3_NH_3_Pb_1−*x*_Mn_*x*_I_3_ PQDs

**Fig. 1:**
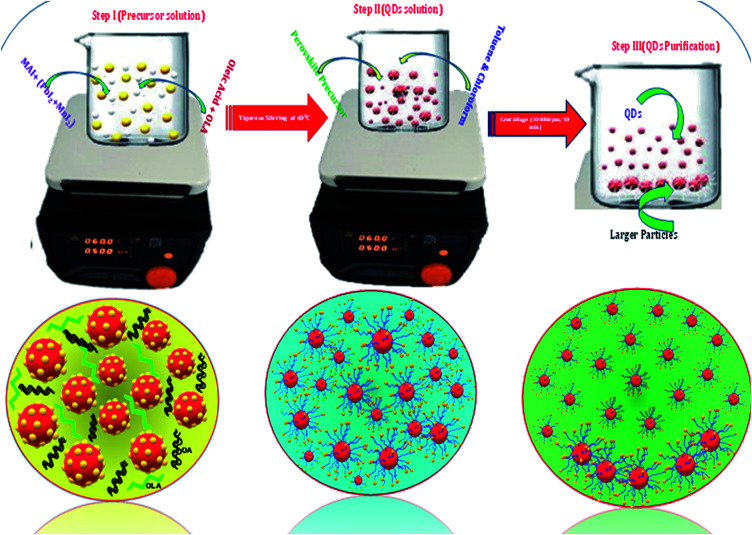
Synthesis process of ligand assisted perovskite quantum dots.

Colloidal CH_3_NH_3_PbI_3_ and CH_3_NH_3_Pb_1−*x*_Mn_*x*_I_3_ QDs were synthesized by following the LARP technique, which is described in the following section. In the typical synthesis of CH_3_NH_3_PbI_3_ perovskite QDs, a mixture of 0.1 mmol (0.0159 gm) CH_3_NH_3_I and 0.1 mmol (0.0461 gm) PbI_2_ was dissolved in 1 ml DMF at 60 °C forming a 0.1 mmol solution. Then, 200 μl of oleic acid and 200 μl of the oleylamine were added to the perovskite solution. 40 μl of precursor perovskite solution was then injected into 6 ml of toluene with vigorous stirring at 70 °C. Along with mixing the perovskite precursor in toluene, bright green emitting nanoparticles were formed within seconds. After centrifugation at 7000 rpm for 10 minutes to discard the larger particles, a red transparent colloidal solution was obtained. A schematic illustration of the synthesis procedure is shown in Fig. 1.

Similarly, for synthesis of CH_3_NH_3_Pb_1−*x*_Mn_*x*_I_3_ QDs, a mixture of 0.01 mmol (0.0159 gm) of CH_3_NH_3_I and (0.1 − *x*) mmol of PbI_2_ and (0.1*x*) [here *x* = 0.05 to 0.60] mmol of MnI_2_ was dissolved in 1 ml of DMF. The rest of the process was the same as the CH_3_NH_3_PbI_3_ QD synthesis. Further details of precursors and conditions are provided in Table S1.† The reason behind the selection of solvent is explained in the ESI.† In the case of iodine-based HPQDs, the selection of solvent is very important because such types of perovskite are very sensitive to the atmosphere and they are also very reactive due to the presence of I^−^ ions. Therefore, a weak (non-polar) solvent is selected which can react very fast with the perovskite material. The reactivity of chloroform is higher than that of toluene due to the high dielectric constant of chloroform (*ε* = 4.81). Hence only chloroform or a mixture of chloroform and toluene is used for CH_3_NH_3_PbI_3_ HPQDs to promote better and fast nucleation. On the other hand, in the case of toluene the reaction kinetics is comparably slow and may lead to the destruction of the QDs during the centrifugation because of the higher sensitivity and instability of iodine-based perovskites.

### Characterization

The XRD of the perovskite QDs was measured using a Bruker D8 powder XRD with Cu Kα radiation over the range of 10 < 2*θ* < 60° with a step size of 0.02 and operating at 40 kV to 40 mA. TEM images and HRTEM patterns were recorded using a JEOL high-resolution transmission electron microscope (HR-TEM) equipped with a LaB6 filament and CCD camera. Samples of different PQD samples for TEM analysis were prepared by casting 10 μl of colloidal solution onto a standard copper grid. The size distribution and particle size of PQDs were obtained from the TEM images and the *d* value was calculated from the HRTEM patterns with ImageJ software. Optical UV-vis absorption spectra were measured using a Hitachi U-2900. Photoluminescence (PL) spectra of PQDs were recorded using an FS-5 Fluorescence Spectrophotometer at 420 nm excitation wavelength in the wavelength range of 500 to 820 nm. The photoluminescence decay time curves were measured using a time correlated single photon counting (TCSPC) system on an FS-5 Fluorescence Spectrophotometer PL system equipped with a 150 W xenon lamp and a 360 nm laser source respectively. The absolute quantum yield (QY) of each PQD sample was determined using a Horiba Jobin Yvon Fluorolog according to the given equation:PLQY (%) = (number of photon emitted/number of photon absorbed) × 100.

For luminescent materials, PLQY characterization is very important for a deep understanding of molecular and light absorbing/emitting properties. Mostly, PLQY is measured using an integrating sphere. From this technique, PLQY can be determined directly. In this tool, a sphere is coated with all reflective surfaces to capture all the light going in and out of the sphere. The PLQY measurement helps to find the fluorescence emission (*E*_c_) and the scattering (*L*_c_) of the sample and also the emission and scattering of a blank *i.e. E*_a_ and *L*_a_ respectively. So with the help of an integral sphere setup, spectral measurements of PLQY can be measured using the given formula;PLQY = ((*E*_c_ − *E*_a_)/(*L*_a_ − *L*_c_)) × 100

## Results and discussion

A series of Mn substituted CH_3_NH_3_PbI_3_ based HPQDs were synthesized by a ligand assisted room temperature approach. The synthesis procedure, the selection of solvent and the thorough description are included in the ESI and the detailed chemical compositions are also given in Table S1.†

**Fig. 2:**
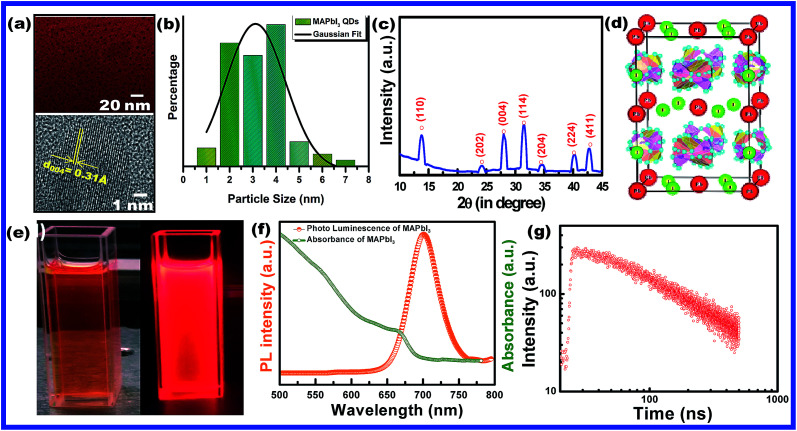
(a) TEM and HR-TEM images (the high resolution image is available in the ESI (Fig. S1†)), (b) particle size-distribution, (c) X-ray diffraction pattern, and (d) corresponding crystal structure of CH_3_NH_3_PbI_3_ (MAPbI_3_) hybrid perovskite quantum dots synthesized *via* a modified low temperature route. (e) Digital photograph of CH_3_NH_3_PbI_3_ PQDs in toluene solution under visible and 365 nm UV light, (f) UV-visible absorbance and photoluminescence spectra and (g) time resolved decay kinetics of CH_3_NH_3_PbI_3_ PQDs.

Fig. 2(a–g) show the phase identification, microscopic characterization, luminescence and time resolved spectroscopy of CH_3_NH_3_PbI_3_ HPQDs. Typical TEM images of the obtained HPQDs showed the homogeneous distribution of tiny spherical HPQDs with an average particle size of ∼3 nm and distribution of 0.03 nm as shown in Fig. 2(a) and (b). The HRTEM image of CH_3_NH_3_PbI_3_ QDs (bottom part of Fig. 2(a)) showed a good crystalline structure with an interplanar distance of 0.31 Å, which corresponds to the (004) plane of CH_3_NH_3_PbI_3_.^18^

Fig. 2(c) shows the XRD pattern of the synthesized CH_3_NH_3_PbI_3_ HPQDs. All diffraction peaks matched well with the reported peaks of CH_3_NH_3_PbI_3_ HPQDs indicating the presence of a tetragonal structure with space group *I*4/*mcm*.^19^ VESTA software was utilized to draw the crystal structure of the obtained HPQDs fused with one Pb^2+^, one CH_3_NH_3_^+^, and three iodine anions in the unit cell as shown in Fig. 2(d). The structural and morphological analyses indicated the formation of a CH_3_NH_3_PbI_3_ perovskite structure in QD form *via* the present synthesis route.

The visual appearance of chloroform/toluene solution of CH_3_NH_3_PbI_3_ HPQDs under a 365 nm UV-light source shows a bright red emission (Fig. 2(e)). The reason behind the selection of solvent is explained in the ESI.†Fig. 2(f) shows the steady-state absorption and photoluminescence (PL) spectra of CH_3_NH_3_PbI_3_ HPQDs. Broad and strong absorption in the visible region and near-infrared region revealed the promising light absorbing quality of QDs. Additionally, the sharp absorption edge of the sample also suggested a direct bandgap nature.^20^ The PL emission of the obtained PQDs was also observed at 700 nm with a high intensity and color purity. The absolute PLQY of CH_3_NH_3_PbI_3_ HPQDs was estimated to be 98%. To the best of our knowledge, it is the highest value reported for CH_3_NH_3_PbI_3_ HPQDs prepared under ambient conditions to date. The PL decay curve can be fitted well with a single exponential function for CH_3_NH_3_PbI_3_ PQDs to obtain an average lifetime of 98.29 ns (Fig. 2(g)). The above discussion indicates an efficient fabrication of red HPQDs with PLQY > 98% and efficient lifetimes.^21^

**Fig. 3:**
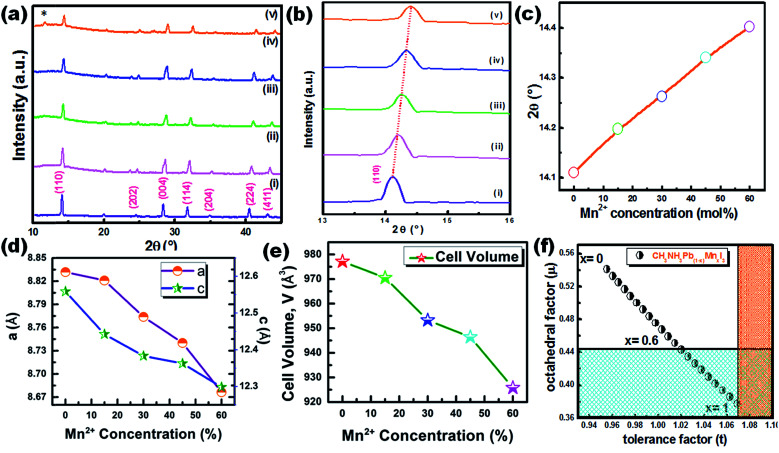
(a) XRD patterns of CH_3_NH_3_Pb_1−*x*_Mn_*x*_I_3_ PQDs for various concentrations of Mn^2+^ as (i) *x* = 0, (ii) *x* = 0.05, (iii) *x* = 0.15, (iv) *x* = 0.45, and (v) *x* = 0.6. (b and c) The higher angle shifting of the enlarged (110) XRD peak position with different Mn concentrations from 0% to 60%. Variation of (d) lattice constants and (e) cell volume of CH_3_NH_3_Pb_1−*x*_Mn_*x*_I_3_ PQDs with different Mn concentrations from 0% to 60%. (f) Theoretical calculations of *t* and *μ* for CH_3_NH_3_Pb_1−*x*_Mn_*x*_I_3_ PQDs for different values of *x*. The white part shows the existence of a perovskite structure while the colored part shows a non-perovskite phase.

The XRD pattern of CH_3_NH_3_Pb_1−*x*_Mn_*x*_I_3_ PQDs with different concentrations of Mn^2+^ substituting Pb^2+^ ions is shown in Fig. 3(a). The XRD pattern indicates the presence of a tetragonal phase for all the synthesized samples even after Mn doping. However, a minimal shifting towards higher 2*θ* was observed for the Mn incorporated perovskites. The higher angle shifting is clear from the variation of the enlarged (110) XRD peak position with different Mn concentrations from 0% to 60% as shown in Fig. 3(b and c). The shifting can be ascribed to the substitution of Mn^2+^ ions with smaller ionic radii compared to Pb^2+^ ions (see Fig. 3(b and c)). The smaller ionic radius of Mn is also expected to cause a reduction of perovskite lattices and the cell volume of the resultant samples as shown in Fig. 3(d and e). According to Vegard's law, the lattice constant and cell volume of the compound have a linear relationship and our experimental data also follow Vegard's law. These results also correlate with previous research studies based on the substitution of Mn with small ionic radii.^19^

**Fig. 4:**
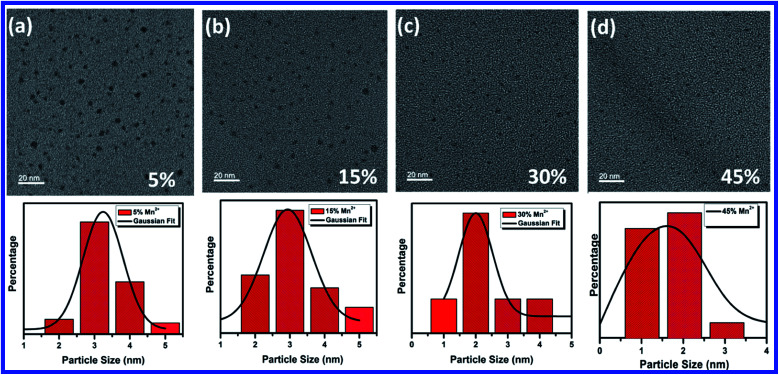
TEM characterization of CH_3_NH_3_Pb_1−*x*_Mn_*x*_I_3_ PQDs with different Mn^2+^ concentration and their corresponding statistical analysis graph of particle size distribution (a) 5% with average particle size 3.5 nm, (b) 15% with average particle size 3 nm, (c) 30% with average particle size 2 nm, and (d) 45% with average particle size 1.5 nm.

TEM images of 5%, 15%, 30% and 45% Mn^2+^ ion doped CH_3_NH_3_Pb_1−*x*_Mn_*x*_I_3_ HPQDs are shown in Fig. 4(a–d) respectively. TEM images of all HPQDs showed spherical dots with an average particle size in the range of ∼1 to 3 nm. The distribution density of particles decreased with the substitution of Mn^2+^ ions with Pb^2+^ ions in CH_3_NH_3_Pb_1−*x*_Mn_*x*_I_3_ PQDs owing to the sensitivity of Mn^2+^ under ambient conditions compared to Pb^2+^.^22^ It is well known that agglomeration in QDs will be less owing to the high surface energy. As we explained in the structural section, according to Vegard's law, the lattice constants and cell volume are directly proportional to the ionic radii of cations present in the compound. Hence, with the substitution of Mn^2+^ ions instead of Pb^2+^ ions the lattice constant is observed to decrease resulting in cell volume contraction. Such reduction in cell volume due to Mn^2+^ substitution in CH_3_NH_3_Pb_1−*x*_Mn_*x*_I_3_ PQDs further results in the reduction of particle size.

**Fig. 5:**
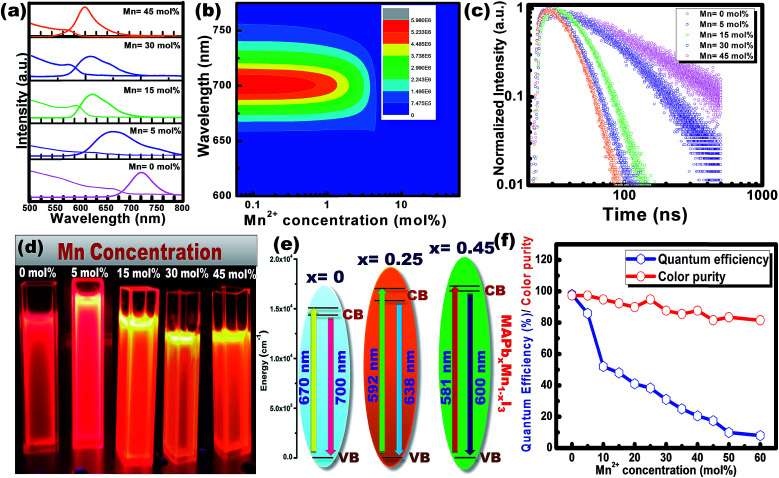
(a) Tunable absorbance and PL spectra of CH_3_NH_3_Pb_1−*x*_Mn_*x*_I_3_ PQDs with the variation of Mn^2+^ concentration, (b) 3-D scan of the variation of PL intensity and wavelength in CH_3_NH_3_Pb_1−*x*_Mn_*x*_I_3_ PQDs with the variation of Mn^2+^ concentration, (c) variation of time resolved decay time and (d) the corresponding digital photo under visible and 365 nm UV light illumination in CH_3_NH_3_Pb_1−*x*_Mn_*x*_I_3_ PQDs with the variation of Mn^2+^ concentration. (e) Energy transfer mechanism from CH_3_NH_3_PbI_3_ to CH_3_NH_3_Pb_0.65_Mn_0.45_I_3_ PQDs. (f) Variation of color purity and PLQY for CH_3_NH_3_Pb_1−*x*_Mn_*x*_I_3_ PQDs with different Mn concentrations.

**Fig. 6:**
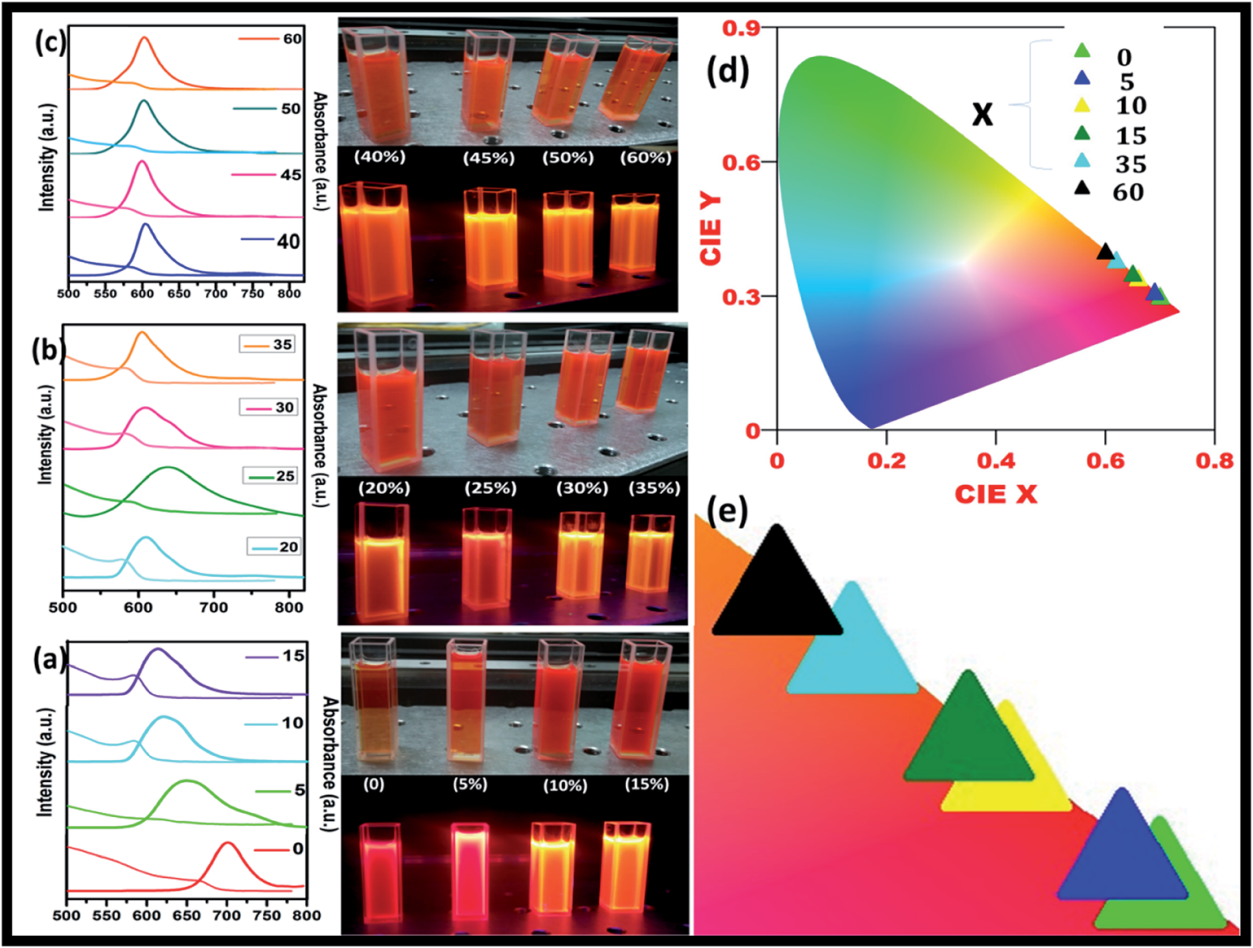
CH_3_NH_3_Pb_1−*x*_Mn_*x*_I_3_ PQDs with different Mn^2+^ concentrations replacing Pb^2+^ in a mixture of toluene and chloroform solutions, (a to c) tunable absorbance and PL spectra of CH_3_NH_3_Pb_1−*x*_Mn_*x*_I_3_ PQDs with 0 to 60% Mn^2+^ concentration and their corresponding digital photo under visible and 365 nm UV light illumination, (d) CIE spectra with color tunability from red to orange and (e) change in color with different Mn^2+^ concentrations in CH_3_NH_3_Pb_1−*x*_Mn_*x*_I_3_ PQDs.

Fig. 5(a) shows the steady-state absorption and PL of CH_3_NH_3_Pb_1−*x*_Mn_*x*_I_3_ HPQDs with the variation of Mn^2+^ from 0 mol% to 45 mol%. Fig. 6(a–c) show the variation of absorption and PL spectra of CH_3_NH_3_Pb_1−*x*_Mn_*x*_I_3_ PQDs with continuous variation of Mn^2+^ ion concentration from 0 mol% to 60 mol%. The absorption spectra showed a blue shift after the replacement of Pb^2+^ with Mn^2+^. The emission peaks of CH_3_NH_3_Pb_1−*x*_Mn_*x*_I_3_ HPQDs also shifted from 700 to 600 nm indicating successful replacement of Pb^2+^ in CH_3_NH_3_PbI_3_ HPQDs.^23*a*^ The changes in the color of HPQDs suggest the successful energy transfer from PQDs to Mn^2+^ dopants in the CH_3_NH_3_Pb_1−*x*_Mn_*x*_I_3_ perovskite samples as shown in Fig. 4(e). Fig. 4(b) displays the 3D scan of the variation of PL emission intensity and wavelength with the increase in Mn^2+^ concentration. PL emission intensity was found to decrease along with a blue shift in color emission as clearly visible in the photographs of the resulting CH_3_NH_3_Pb_1−*x*_Mn_*x*_I_3_ HPQD colloidal solutions (Fig. 5(d) and Fig. 6(a–c)). Furthermore, to better enable the comparison of color variation, the influence of Pb^2+^ to Mn^2+^ cation exchange on the color coordinates of CH_3_NH_3_Pb_1−*x*_Mn_*x*_I_3_ PQDs is shown in the ESI† and Fig. 6(d and e).

The PL emission spectra reveal an interesting broadening for Mn content along with an obvious blue shift due to the combined effect of quantum confinement and Mn incorporation. It is well known that agglomeration in QDs will be less owing to the high surface energy. Moreover, as the ionic radius of Mn^2+^ ions is lower than that of Pb^2+^ ions, the lattice volume starts to shrink with the incorporation of Mn^2+^ ions into the CH_3_NH_3_PbI_3_ host. And hence the particle size starts to decrease with the incorporation of Mn^2+^ ions in place of Pb^2+^ ions as is clear from the TEM image and corresponding statistical distribution. As the particle size starts to decrease from approximately 4 nm to 1.5 nm, the quantum confinement plays a role for the blue shifting of PL emission. Fig. 6(a) shows the steady-state absorption and PL of CH_3_NH_3_Pb_1−*x*_Mn_*x*_I_3_ HPQDs with the variation of Mn^2+^ from 0 mol% to 60 mol%. The absorption spectra showed a blue shift after the replacement of Mn^2+^ in Pb^2+^ sites. However, careful examinations of PL emission spectra reveal an interesting broadening with the increase in Mn concentration as shown in Fig. S3.† The emission spectra of CH_3_NH_3_Pb_1−*x*_Mn_*x*_I_3_ with *x* = 0, 0.05, 0.15, 0.30 and 0.60 are shown in Fig. S3(i–v)† respectively. The emission spectra of CH_3_NH_3_PbI_3_ shows a broad band around 700 nm which is blue shifted compared to its bulk counterpart (Fig. S2†). With the increase in Mn^2+^ concentration, the peak position blue shifted due to the quantum confinement effect as discussed earlier. Hassan *et al.* have also observed similar results to ours.^23*b*^ However, the PL emission peak can be deconvoluted into two distinguished peaks centred at 634 and 600 nm. According to the results of Hassan *et al.*, in the present research, 634 nm emissions can be assigned to the characteristic emission of the CH_3_NH_3_PbI_3_ perovskite host. Zou *et al.*^23*c*^ have reported the dominant broad emission band peaking at ∼600 nm of the perovskite host, which can be readily ascribed to the ^4^T_1_ → ^6^A_1_ transition of Mn^2+^ doped in CH_3_NH_3_Pb_1−*x*_Mn_*x*_I_3_. When the Mn^2+^ concentration is more than 30%, the contribution from Mn^2+^ is dominant over the host perovskite and hence at 60% Mn^2+^ doping only a 600 nm peak is visible. Furthermore, CH_3_NH_3_Pb_1−*x*_Mn_*x*_I_3_ (*x* = 0) and CH_3_NH_3_Pb_1−*x*_Mn_*x*_I_3_ (*x* = 0.3) PQDs exhibit almost the same PL excitation spectra when monitoring their excitonic or Mn^2+^-related emissions. The similar excitation features suggested a clear energy transfer from the CH_3_NH_3_PbI_3_ host Mn^2+^.^23^

Time-resolved PL spectroscopy was used to correlate the variation of the PL emission efficiency of the CH_3_NH_3_Pb_1−*x*_Mn_*x*_I_3_ (*x* = 0 to 0.60) as shown in Fig. 5(c) and S4(a–c).†

For CH_3_NH_3_PbI_3_ and CH_3_NH_3_Pb_0.95_Mn_0.05_I_3_ PQDs, single exponential PL decay was observed reflecting the homogeneous distribution of the elements. The result was similar to PL decay in Mn^2+^: ZnSe and Mn^2+^:CdS/ZnS nano-crystals as reported earlier.^24,25^ However, for more than 5% Mn^2+^ doping, the PL decay curve could be fitted well with a biexponential function. For more than 5% Mn^2+^ doping, the PL decay curve could be fitted well with a bi-exponential function as follows1
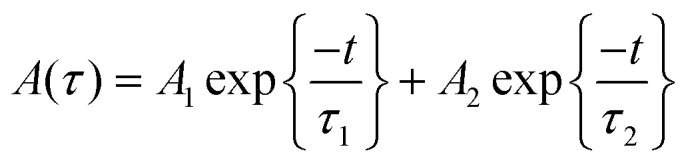
where, *A*, *A*_1_, *A*_2_ and, *t* represent constants and time, respectively, while *τ*_1_ and *τ*_2_ represent short-lived and long-lived decay lifetimes that originated from the trap-assisted recombination and carrier radiative recombination, separately. The average lifetime of Mn^2+^ doped PQDs can be measured as per the formula

2 *τ*_av_ = *A*_1_*τ*_1_^2^ + *A*_2_*τ*_2_^2^/*A*_1_*τ*_1_ + *A*_2_*τ*_2_

The average lifetime of 0 to 60% Mn^2+^ doped CH_3_NH_3_PbI_3_ PQDs gradually decreases as the emission shifts to a shorter wavelength because of the increased content of Mn^2+^ (Mn^2+^ has a lower atomic no. than Pb^2+^). CH_3_NH_3_PbI_3_ and CH_3_NH_3_Pb_0.95_Mn_0.05_I_3_ PQDs have a longer lifetime that can be attributed to the lower non-radiative energy transfer, trap-defects and surface states of the QDs. The decrease in *τ*_av_ with increase in Mn^2+^ concentration in CH_3_NH_3_Pb_1−*x*_Mn_*x*_I_3_ PQDs is shown in Fig. S4.†

The maximum color purity (details are given in the ESI†) was estimated to be 97% for CH_3_NH_3_PbI_3_ and it continually decreased with the elevation of the doping concentration of Mn^2+^ (Fig. 5(f)). The effect on PLQY of Mn^2+^ concentration in CH_3_NH_3_PbI_3_ PQDs is shown in Fig. 5(f). The total external PLQY of CH_3_NH_3_PbI_3_ and CH_3_NH_3_Pb_0.95_Mn_0.05_I_3_ was observed to be 98% and 86%, respectively, and then decreased with further increase in Mn^2+^ concentration. Promising PLQY has been obtained at even low concentrations of Mn^2+^ (up to 20%). It is the highest concentration when compared to analogous results for Mn^2+^ doped nanocrystals.^26–29^ The absolute PLQY, average lifetime and color purity of PQDs with different concentrations of MnI_2_ are summarized in Table 1 and a plot between the PLQY and color purity is also shown in Fig. S5.†

**Table 1:** Summary of different PQDs including PLQY, average lifetime, and color purity

PQDs	PLQY (%)	*τ*_1_ (ns)	(A_1_)	*τ*_2_ (ns)	(A_2_)	Average lifetime (ns)	Color purity (%)
CH_3_NH_3_PbI_3_	98.00	98.29	2.28	—	—	98.29	85.28871
CH_3_NH_3_Pb_0.90_Mn_0.05_I_3_	86.00	69.09	2.31	—	—	69.09	99.12048
CH_3_NH_3_Pb_0.90_Mn_0.10_I_3_	52.00	30.26	2.29	44.50	4.13	36.48	96.53949
CH_3_NH_3_Pb_0.85_Mn_0.15_I_3_	48.00	17.53	1.86	29.83	2.12	24.64	95.38515
CH_3_NH_3_Pb_0.80_Mn_0.20_I_3_	41.00	15.39	2.32	26.84	3.37	21.44	94.05758
CH_3_NH_3_Pb_0.75_Mn_0.25_I_3_	38.30	13.32	1.77	21.10	1.00	19.05	99.12048
CH_3_NH_3_Pb_0.70_Mn_0.30_I_3_	31.00	13.13	1.86	20.93	2.65	17.28	91.68279
CH_3_NH_3_Pb_0.65_Mn_0.35_I_3_	25.00	13.17	1.92	18.34	1.13	16.81	91.68279
CH_3_NH_3_Pb_0.60_Mn_0.40_I_3_	20.50	11.47	3.24	15.23	1.63	14.20	89.42367
CH_3_NH_3_Pb_0.55_Mn_0.45_I_3_	17.50	12.20	1.84	14.93	1.15	14.00	85.28871
CH_3_NH_3_Pb_0.50_Mn_0.50_I_3_	10.0	11.14	3.24	13.13	1.63	12.53	87.28919
CH_3_NH_3_Pb_0.40_Mn_0.60_I_3_	8.0	11.21	1.92	12.23	1.13	11.88	85.28871

It is evident that with the Mn^2+^ ion substitution in CsMn_1−*x*_Pb_*x*_I_3_ PQDs, the PLQY is observed to drastically reduce from 98% to 8%. The reason for such a decrease could be attributed to the change in the energy band structure due to Mn^2+^ ion substitution. In the case of manganese ion substitution, the electrons are distributed between t^g^_2_ and e^g^ bands according to crystal field stabilization theory. However, for Mn^2+^ ions the unpaired electrons in the d orbital remain distributed in the low energy t^g^_2_ band leaving behind empty e_g_ bands. In the case of excitation, the electrons in the t^g^_2_ band may get excited to the e_g_ band, which is essentially a non-radiative transformation owing to its low energy difference, and this mechanism is shown in Fig. S6.† In such a scenario, the excitation of electrons leads to a radiation quenching effect. Thus low photo luminescence quantum yields (PLQYs) and reduced lifetimes are observed in the case of Mn^2+^ ion substitution.

**Fig. 7:**
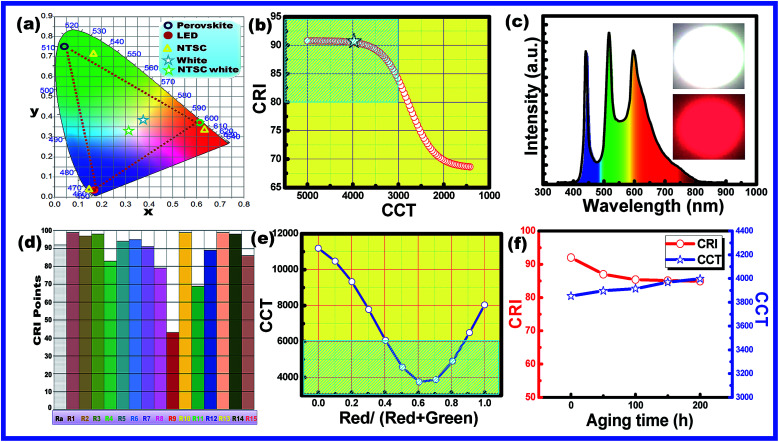
(a) Chromaticity coordinates of the blue LED, and green and red PQDs in the CIE diagram (*XYZ* colour space). The green star indicates the NTSC white coordinates. The blue star represents the colour points of the fabricated W-LED. The yellow triangles are the colour coordinates of NTSC red chromaticity. (b) Plot of the CCT–CRI curve estimated from simulation results. The black star represents the point for the fabricated W-LEDs in the present case. (c) Electroluminescence spectra and (d) various CRI values of the fabricated warm white light LEDs made of a blue LED chip and green and red PQDs. The inset shows a digital image of the prototype warm-white LED. (e) Tuning of CCT values of the warm white light LEDs made of a blue LED chip and green and red PQDs with the variation of red and green PQDs. (f) Variation of CRI and CCT of the warm white light LEDs made of the blue LED chip and green and red PQDs with aging time unto 200 h.

The obtained CH_3_NH_3_Pb_1−*x*_Mn_*x*_I_3_ PQDs were utilized to fabricate LED devices. In the present case, the perovskite emitters were integrated as color converting layers combined with blue LEDs and the color parameters of the obtained W-LEDs are shown in Fig. 7. From the CIE chromaticity diagram (Fig. 7(a)) it is clear that the connection lines of the color point of blue LED with green and red PQDs always pass through the white emission and hence the combination can produce white light consistently. Therefore, white light emissions were simulated by the linear combination of the emission of green and red PQDs with blue LEDs. Color correlated temperature (CCT) and color rendering index (CRI) values of the corresponding white emission were evaluated with the help of methods reported earlier.^30,31^

The evaluated data were used to plot a CCT–CRI diagram as shown in Fig. 7(b). The simulation results indicated that warm white light with CCT < 5000 K and CRI > 80 (blue shaded area) can be simply realized in the current combination. In the present simulation, a high CRI of 92 was obtained at a CCT of 5100 K and the lowest CCT value of 2900 K with a CRI of 80 was obtained. The simulation results indicated that the present combination can produce an efficient warm white light with a promising CRI.^32^ Therefore, a series of WLEDs were fabricated by encapsulating a blue InGaN LED chip with various amounts of green and red PQDs to validate the simulation results. The electroluminescence (EL) spectra of the fabricated WLED are shown in Fig. 7(c). The inset of Fig. 7(c) presents the fabricated WLED with high brightness. WLEDs with chromaticity coordinates at (0.34, 0.37), a CRI of 91 and a CCT of 4000 K were obtained and are shown as star points in Fig. 7(a). Different color rendering parameters for the fabricated WLEDs are shown in Fig. 7(d). The figure indicated the promising CRI nature of the obtained WLEDs. The CCT values can be tuned with the change in red to green ratios from cool light (11 000 K) to warm light (3800 K) as shown in Fig. 7(e).

The shaded region in Fig. 7(e) confirmed the presence of warm white light emission from the present materials by controlling the amount of green and red emitting PQDs.^33^ The results are consistent with the simulation results. The combination of a blue LED chip and green and red PQDs results in efficient warm white light (4000 K) with a high CRI (91) along with an improved stability since only ∼10% of the initial intensity is lost after 200 h under accelerated aging conditions (85 °C and 85% relative humidity) as can be observed in Fig. 7(f). From the above discussion it is clear that, the present PQDs can act as an efficient red emitting material for warm white light emitting applications.

## Conclusion

In conclusion, we have offered an inexpensive and speedy room temperature synthesis method for the fabrication of CH_3_NH_3_Pb_1−*x*_Mn_*x*_I_3_ PQDs with spherical shape and a particle size of ∼1.5 to 3 nm. Absorbance and PL emission studies show that with increasing Mn^2+^ substitution of Pb^2+^, in CH_3_NH_3_PbI_3_, the PQDs produce an emission colour varying from red to orange due to efficient energy transfer from QDs to Mn^2+^ ions. The unsubstituted PQDs showed PLQYs of nearly 98% while with 5% Mn^2+^ substitution the PLQY decreases to 86% and further declines with increasing concentration of Mn^2+^. The average lifetime of pure (CH_3_NH_3_PbI_3_) and PQDs with very low concentration of Mn^2+^ was observed to be longer than that of the PQDs with higher Mn^2+^ concentration. The fabricated series of WLEDs combining a blue InGaN LED chip with various amounts of green and red PQDs produced warm white light with CCT < 5000 K and CRI > 80. The best WLED with chromaticity coordinates at (0.34, 0.37), a CRI of 91 and a CCT of 4000 K was obtained with an improved stability since only ∼10% of the initial intensity is lost after 200 h under accelerated aging conditions (85 °C and 85% relative humidity). These results confirm the commercial usability of the present PQDs for general lighting applications as environment friendly QD-LEDs and backlight systems.

## Conflicts of interest

There are no conflicts to declare.

## Supplementary Material

NA-001-C9NA00330D-s001
