# Supplementary material for: Rapid synthesis of hybrid methylammonium lead iodide perovskite quantum dots and rich MnI2 substitution favouring Pb-free warm white LED applications
Source: Nanoscale Adv. 2019 Jun 7;1(8):2999–3008. doi: 10.1039/c9na00330d (PMC9419008; doi:10.1039/c9na00330d)
Supplement: NA-001-C9NA00330D-s001 [file NA-001-C9NA00330D-s001.pdf]

Electronic Supplementary Material (ESI) for Nanoscale Advances.
This journal is © The Royal Society of Chemistry 2019

Rapid synthesis of hybrid methylammonium lead iodide perovskite quantum dots and rich MnI_2 substitution favouring Pb- free warm white LED applications

Rajan Kumar Singh[a,b], Sudipta Som[b], Somrita Dutta[c], Neha Jain[a], Mei-Tsun Kuo[b], Jai Singh[a], Ranveer Kumar[a,*] and Teng-Ming Chen [c,*]

[a] Department of Physics, Dr. Harisingh Gour Central University, Sagar, 470003, M. P., India

[b] Department of Chemical Engineering, National Taiwan University, Taipei, Taiwan, ROC

[c]Department of Applied Chemistry, National Chiao Tung University, 1001 University Road, Hsinchu 30010, Taiwan

----

*To whom all correspondence should be addressed.

Email: tmchen@mail.nctu.edu.tw, ranveerssi@yahoo.com,

Tel: +88635731695, Fax: +88635723764.

Table S1 Detailed composition and amount of ingredients required for the synthesis of $CH_3NH_3PbI_3$ based HPQDs.

S.N	PQDs	CH_3NH_3I (gm)	PbI_2 (gm)	MnI_2 (gm)	Oleic Acid (µl)	Olelyla mine (µl)	Perovskite Precursor (µl)	Equal mixture of Toluene and chloroform(ml)
1	$CH_3NH_3PbI_3$	0.0159	.0461	0	200	200	40	6
2	$CH_3NH_3Pb_{0.90}Mn_{0.05}I_3$	0.0159	.0438	.0015	200	200	40	6
3	$CH_3NH_3Pb_{0.90}Mn_{0.10}I_3$	0.0159	0.415	.0031	200	200	40	6
4	$CH_3NH_3Pb_{0.85}Mn_{0.15}I_3$	0.0159	0.392	.00464	200	200	40	6
5	$CH_3NH_3Pb_{0.80}Mn_{0.20}I_3$	0.0159	.0369	.0062	200	200	40	6
6	$CH_3NH_3Pb_{0.75}Mn_{0.25}I_3$	0.0159	.0346	.0077	200	200	40	6
7	$CH_3NH_3Pb_{0.70}Mn_{0.30}I_3$	0.0159	.0323	.0093	200	200	40	6
8	$CH_3NH_3Pb_{0.65}Mn_{0.35}I_3$	0.0159	0.03	.0108	200	200	40	6
9	$CH_3NH_3Pb_{0.60}Mn_{0.40}I_3$	0.0159	.0278	.0124	200	200	40	6
10	$CH_3NH_3Pb_{0.55}Mn_{0.45}I_3$	0.0159	.0254	.014	200	200	40	6
11	$CH_3NH_3Pb_{0.50}Mn_{0.50}I_3$	0.0159	.0231	.0155	200	200	40	6
12	$CH_3NH_3Pb_{0.40}Mn_{0.60}I_3$	0.0159	.0184	.01854	200	200	40	6

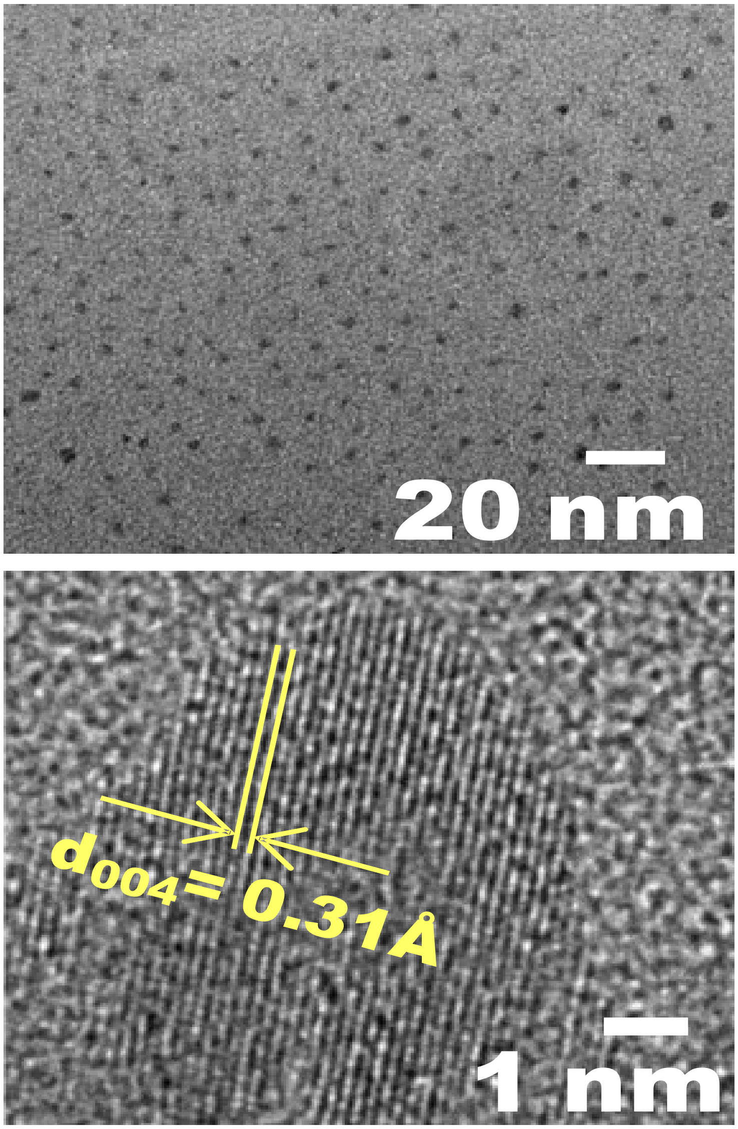

Figure S1: TEM and HR-TEM imageof $CH_3NH_3PbI_3$ ($MAPbI_3$) hybrid perovskite quantum dots synthesized via a modified low temperature route.

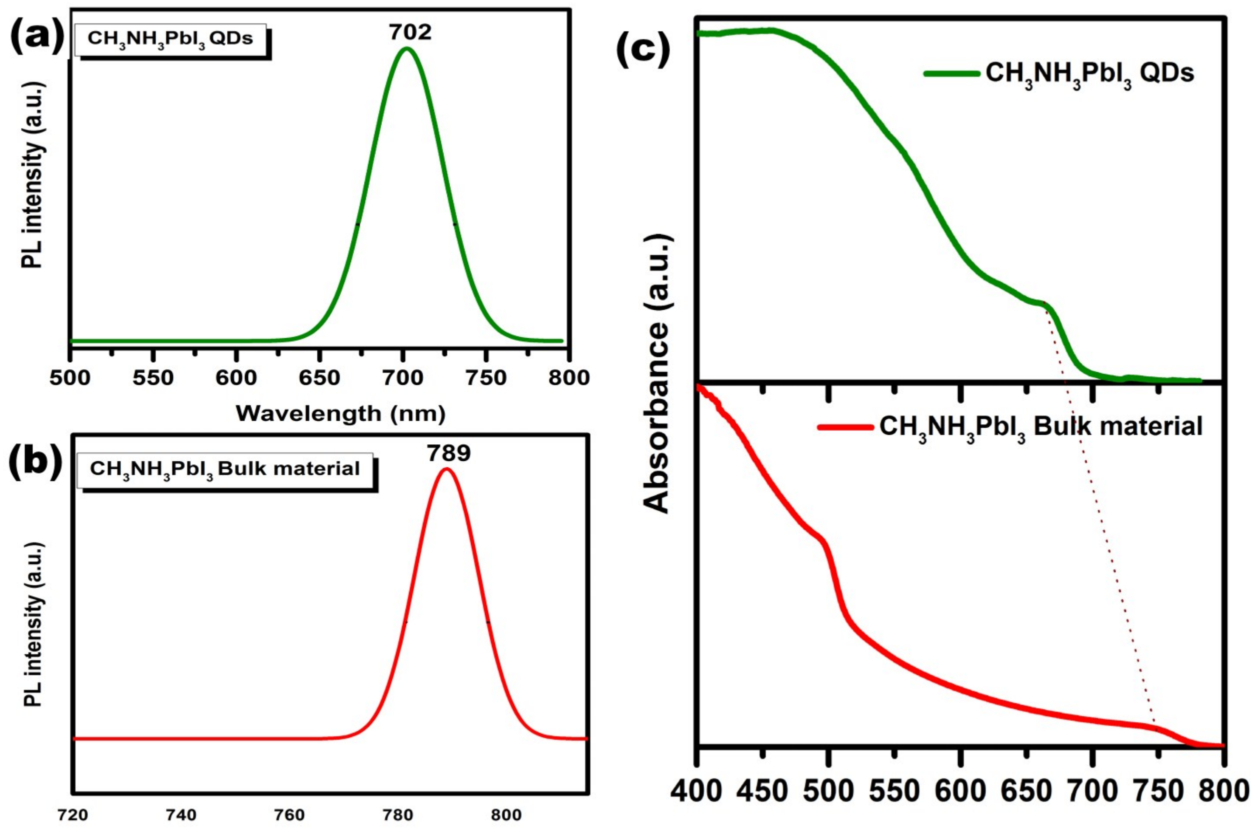

Figure S2: Comparision of bulk and PQDs of $CH_3NH_3PbI_3$ (a and b) PL emission of $CH_3NH_3PbI_3$ QDs and bulk material respectively and (c) UV-visible absorbance spectra of $CH_3NH_3PbI_3$ QDs and bulk material.

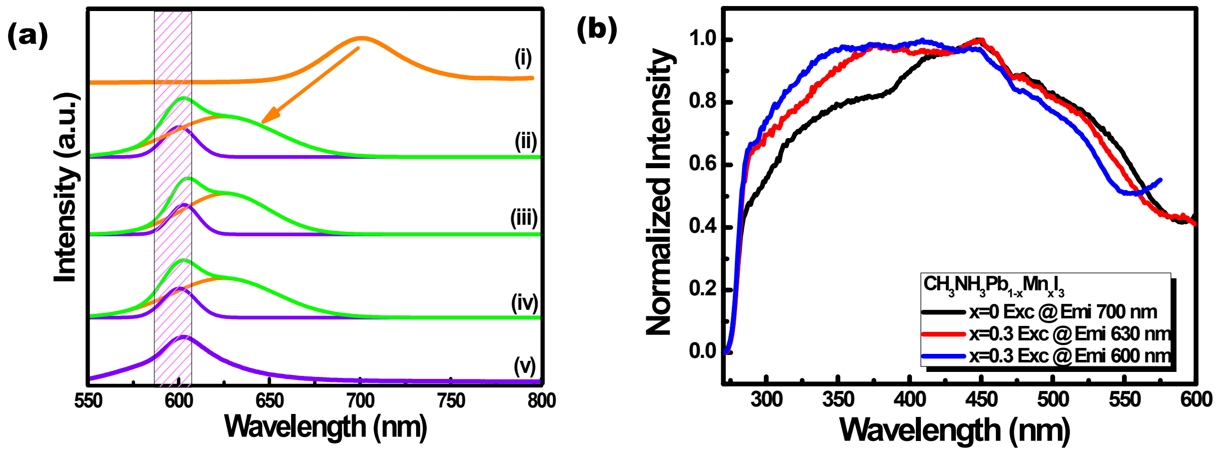

Figure 3: (a) Variation of PL emission spectra from 0% to 60% and decomposition of PL peaks into two Gaussian curves, and the (b) comparison of PL excitation spectra for pure and 30% doped $CH_3NH_3Pb_{1-x}Mn_xI_3$ PQDs by monitoring the excitonic and Mn^{2+} related emissions.

Color coordinate diagram:

The influence of Pb^{2+} to Mn^{2+} cation exchange on colorcoordinates of $CH_3NH_3Pb_{1-x}Mn_xI_3$ PQDs is shown in the commission International deL'Eclairage (CIE) chromaticity diagrams to better enable the comparison of color variation (Fig. S2(d)). With the gradual substitution of Pb^{2+} by Mn^{2+}, the emission color changed from red/pink to orange for the 5 to 60% Mn^{2+} doped PQDs and CIE co-ordinates lies between (x= 0.67 to 0.60 and y= 0.30 to 0.40).

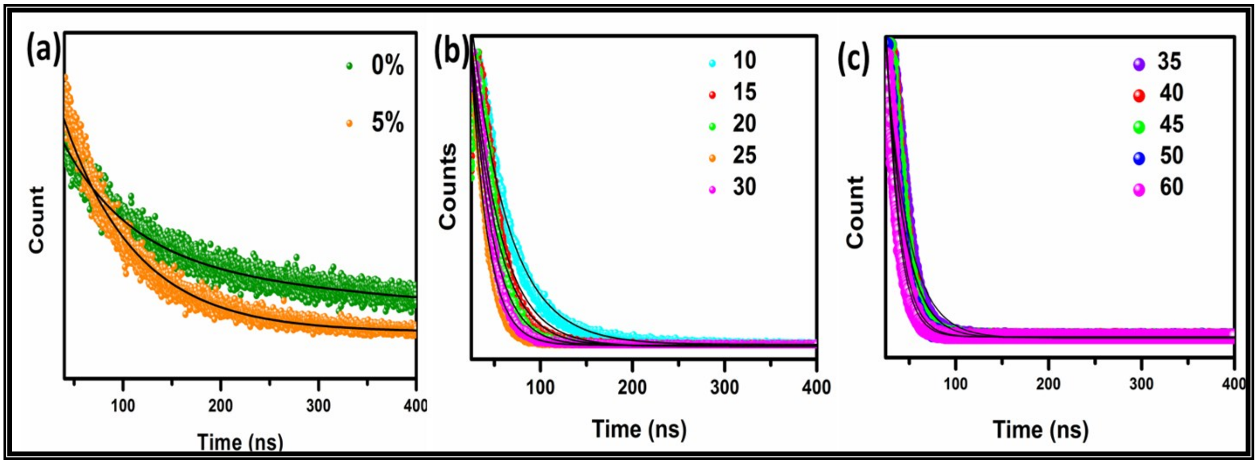

Figure S4: PL life time deacy curves (a)TRPL of pure $CH_3NH_3PbI_3$ and 5% Mn^{2+} doped PQDs fitted with mono-exponential fitting (b) TRPL of 10 – 30% and (c) 35-60% Mn^{2+} incorporated emplace of Pb^{2+} in $CH_3NH_3Pb_{1-x}Mn_xI_3$ PQDs with dual-exponential fitting, (d) graph between average life-time and Mn^{2+} concentration in $CH_3NH_3Pb_{1-x}Mn_xI_3$ PQDs

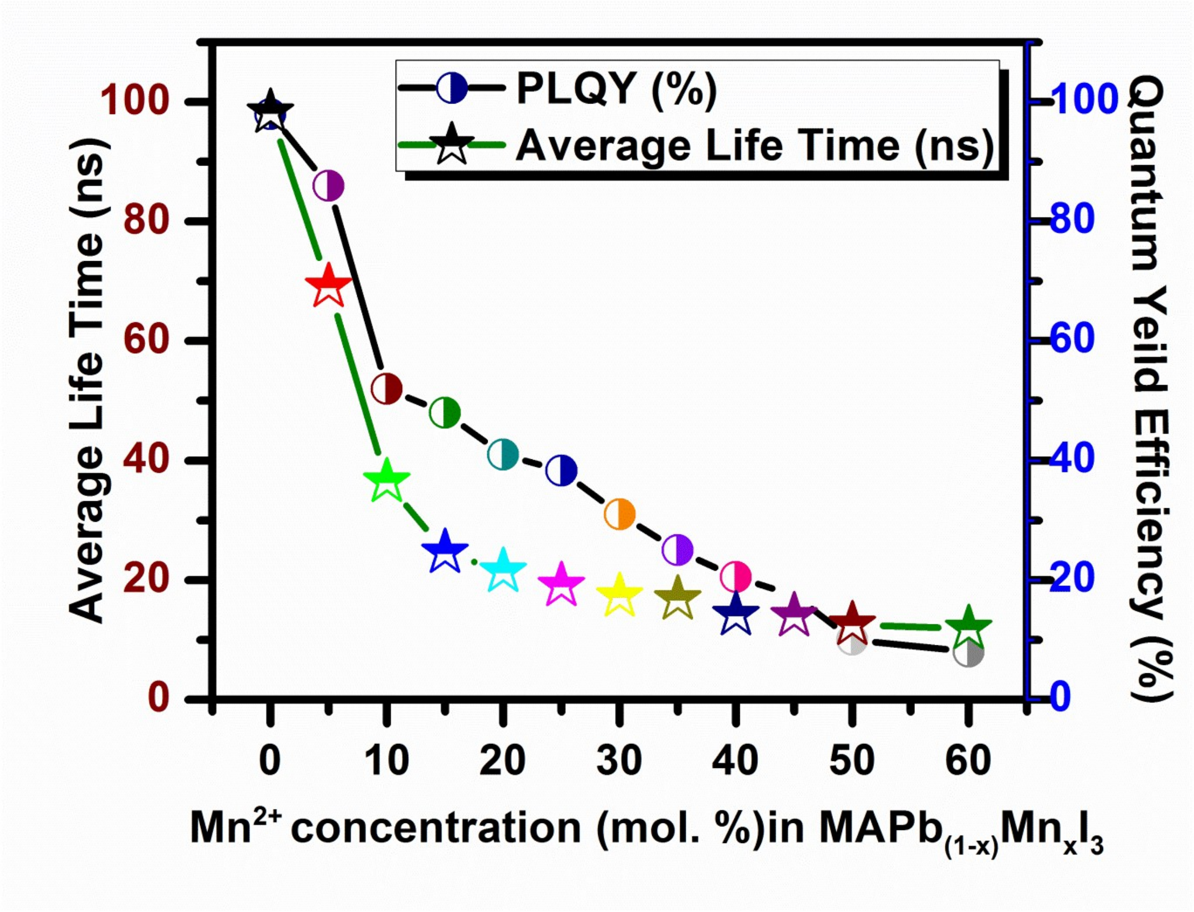

Figure S5: Graph between PLQY and average life-timefor different concentration of Mn^{2+} concentration in $CH_3NH_3Pb_{1-x}Mn_xI_3$ PQDs

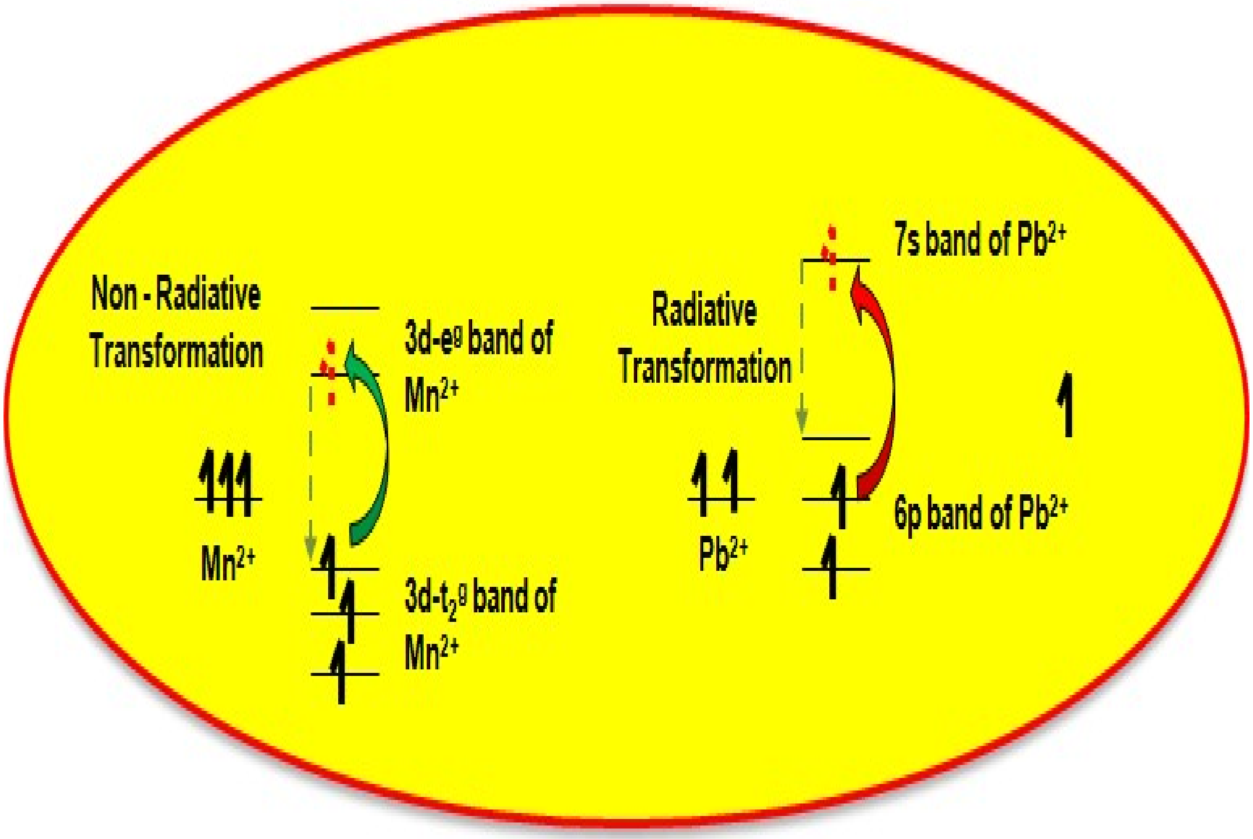

Figure S6: Transition mechnism in perovskite with Mn incorporation
